# Macrocyclic Spermidine Alkaloids from *Androya decaryi* L. Perrier

**DOI:** 10.3390/molecules18043962

**Published:** 2013-04-04

**Authors:** Anne-Cécile Le Lamer, Nehal Ibrahim, Frédéric Manjary, Sonia Mallet-Ladeira, Cynthia Girardi, Alexis Valentin, Nicolas Fabre, Claude Moulis

**Affiliations:** 1 Université de Toulouse; UMR 152 UPS-IRD (Pharmacochimie et Pharmacologie pour le Développement-PHARMA DEV), F-31062 Toulouse Cedex 9, France; 2 Laboratoire de Chimie de la Faculté des Sciences, Université de Toliara, BP 185 Maninday Toliara 602, Madagascar; 3 Institut de Chimie de Toulouse, Université de Toulouse, UPS, FR2599, 118 route de Narbonne, F-31062 Toulouse Cedex 9, France

**Keywords:** *Androya*, Scrophulariaceae, spermidine alkaloids

## Abstract

Three new spermidine alkaloids and two known compounds were isolated from the leaves of *Androya decaryi*. Their structures were elucidated on the basis of their spectroscopic data (NMR and mass spectrometry), by X-Ray diffraction and by comparison with literature values. Evaluation of the *in vitro* antiplamosdial properties of the isolated compounds revealed they did not possess any significant activity.

## 1. Introduction

*Androya decaryi* L. Perrier (Scrophulariaceae) [1] is an evergreen shrub endemic to the southwest of Madagascar. Traditionally used in folk medicine, the decoction of the aerial parts is used to treat yellow fever and malaria-like symptoms (according to local ethnopharmacological investigations). Considering the need for new antiplasmodial compounds due to the emergence of resistant strains of *Plasmodium falciparum* [2], and the fact that natural products and phytomedicines are still promising sources of antimalarial agents [3,4,5], the aerial parts of *A. decaryi* were phytochemically investigated.

Herein, we report the isolation and structural identification of three new macrocyclic spermidine alkaloids **1**-**2a/b**, along with a lignan **3** and (*E*)-cinnamamide (**4**) from the leaves *A. decaryi*, as well as the evaluation of their *in vitro* antiplasmodial activity. To the best of our knowledge, this is the first report on phytochemistry of the genus *Androya* and on spermidine alkaloids from a plant belonging to the Scrophulariaceae.

## 2. Results and Discussion

**Figure 1:**
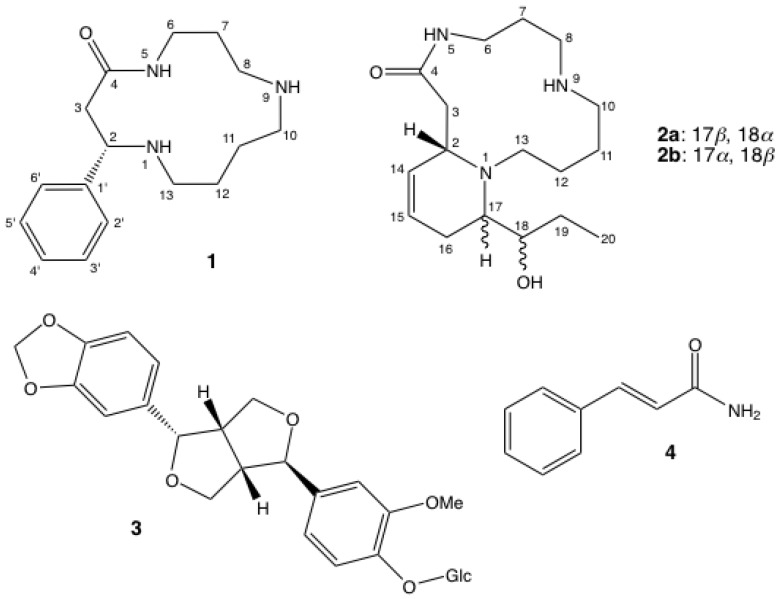
Structures of compounds **1–4**.

Since alkaloids have been detected by TLC in the leaves of *A. decaryi*, a leaf alkaloid extract was studied. Compounds **1** and **2a** (Figure 1) were isolated from the crude alkaloid fraction. Minor alkaloids were also detected; a second extraction was therefore performed on a larger amount of *A. decaryi*, leading to the isolation of the alkaloid **2b** and compounds **3** and **4** (Figure 1).

Compound **1**, a yellow oil, had the molecular formula C_16_H_25_N_3_O as established by HR-ESIMS (*m/z* 276.2078 [M+H]^+^). The 16 carbon signals (Table 1) were identified with *J*-modulated ^13^C-NMR spectrum as eight sp^3^ methylenes, one sp^3^ methine, five sp^2^ methines and two sp^2^ quaternary carbons, including one amide carbonyl. The ^1^H-NMR and HSQC spectra displayed signals for three pairs of aliphatic methylene protons between *δ*_H_ 1.40 and 1.85, five pairs of deshielded methylene protons between *δ*_H_ 2.33 and 3.74, one deshielded methine (*δ*_H_ 4.01), and a monosubstituted benzene ring. These data were consistent with a 13-membered cyclic spermidine alkaloid bearing an aromatic ring. Analysis of COSY and HMBC spectra, measurement of a negative optical rotation (${\left[\alpha \right]}_{D}^{25}$ = −13 (c 3.0, MeOH), and comparison with literature values indicated that Compound **1** was (–)-(2*S*)-2-phenyl-1,5,9-triazacyclotridecan-4-one [6]. Although this alkaloid has been used as a building block for the synthesis of other natural spermidine alkaloids [6,7,8], it is reported here for the first time as a natural product.

**Table 1:** ^1^H and ^13^C-NMR data for Compound **1** in CDCl_3_.

	*δ*_H_ (*J* in Hz) ^a^	*δ*_C_
2	4.01 dd (11.5, 2.9)	60.0
3	2.48 dd (15.0, 2.9); 2.56 dd (15.0, 11.5)	45.0
4	-	171.7
5	8.57 bs	-
6	3.19 dt (13.7, 5.5); 3.70 dt (13.7, 5.2)	39.6
7	1.80–1.85 m	27.8
8	2.86 dt (12.1, 5.0) 2.96 dt (12.1, 5.6)	49.7
10	2.74–2.75 m	49.1
11	1.55–1.61 m; 1.74–1.80 m	27.9
12	1.40–1.49 m; 1.63–1.69 m	27.6
13	2.33 ddd (12.5, 8.8, 1.9); 2.52–2.56 m	45.8
1’	-	142.7
2'/6'	7.23–7.27 m	126.4
3'/5'	7.30–7.36 m	128.7
4'	7.23–7.27 m	127.3

**Figure 2:**
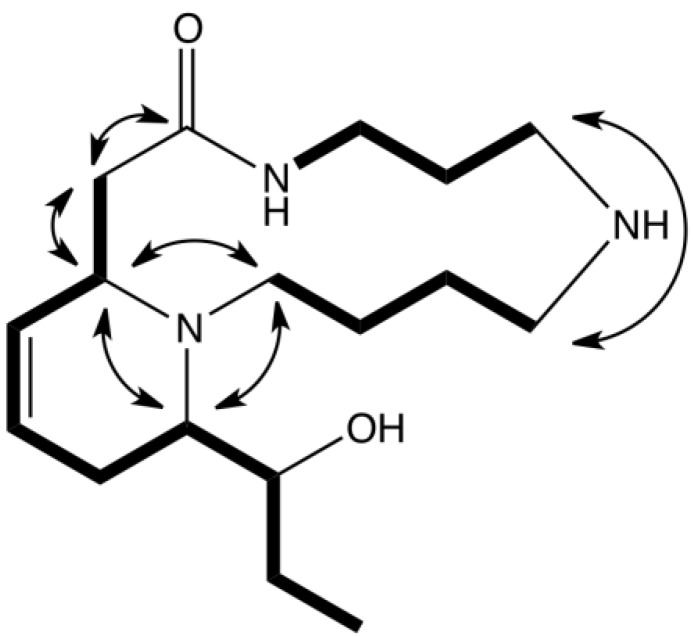
Selected COSY (**bold**) and HMBC correlations of compound **2a**.

**Figure 3:**
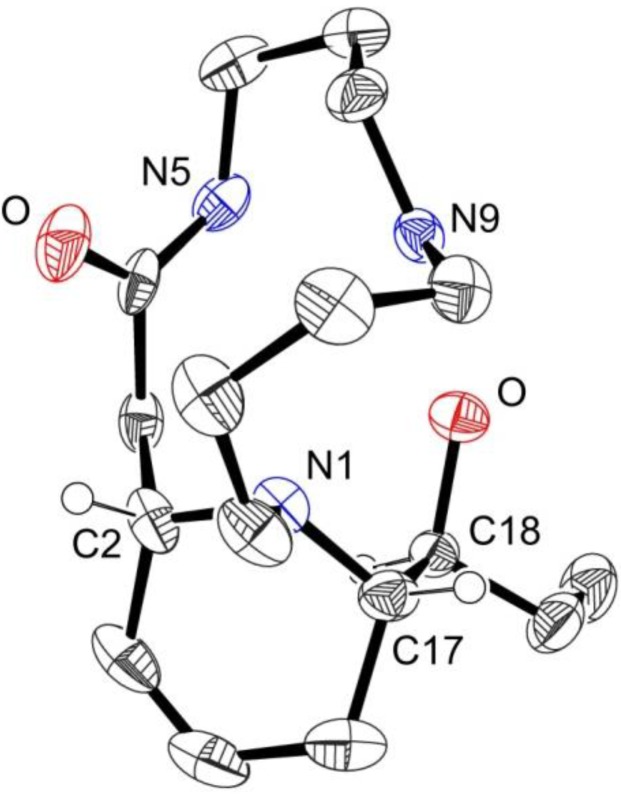
Perspective drawing of the X-Ray structure of compound **2a** hydrochloride. Thermal ellipsoids are set at the 50% probability level. The solvent molecule, the chloride counter anion and all hydrogen atoms, except those on the chiral carbons, have been omitted for clarity.

Compound **2a** was obtained as colorless crystals (CH_2_Cl_2_). In the HR-ESIMS spectrum, it exhibited a quasi-molecular ion at *m/z* 310.2501 [M+H]^+^, establishing a molecular formula of C_17_H_31_N_3_O_2_. The molecular formula and the general framework of the ^1^H and ^13^C-NMR spectra (Table 2) indicated that compound **2a** was also a macrocyclic lactam spermidine alkaloid. The *J*-modulated ^13^C-NMR and HSQC spectra displayed signals for an amide carbonyl carbon at *δ*_C_ 173.2, two methylenes adjacent to an amide function at *δ*_C_ 37.4 (CONH-CH_2_) and *δ*_C_ 41.9 (CH_2_-CONH), three nitrogenated CH_2_ (*δ*_C_ 45.8, 46.3 and 54.4), three methylenes (*δ*_C_ 22.2, 22.5 and 26.9) and one methine carbon at *δ*_C_ 56.5. These signals were consistent with a macrocyclic spermidine moiety and the partial structure was confirmed by analysis of the ^1^H-NMR spectroscopic data (Table 2), and COSY and HMBC correlations (Figure 2). Moreover, two additional methylene carbons (*δ*_C_ 20.5, 28.9), one methyl at *δ*_C_ 10.7, and four deshielded methine, including one oxygenated (*δ*_C_ 73.0) and two olefinic methines at *δ*_C_ 123.7 and 126.6, were observed. The ^1^H-NMR data and COSY correlations supported the presence of an ethyl moiety adjacent to an oxymethine and a double bond (*δ*_H_ 5.59, d, *J* = 10 Hz and 5.79–5.84, m). The connectivity between H-2/H-14, H-14/H-15, H-15/H_2_-16, H_2_-16/H-17, H-17/H-18, H-18/H_2_-19 and H_2_-19/H-20 was evident from COSY correlations suggesting that an unsaturated and hydroxylated chain was linked to the macrocyclic lactam through C-2. Further HMBC correlations revealed that C-17 was also connected through N-1 to the macrocyclic lactam (C-17/H-2 and C-17/2H-13). This was in accordance with the downfield chemical shift of C-17 (*δ*_C_ 63.5). The structure of **2a** was finally confirmed by single-crystal X-ray diffraction (Figure 3). Moreover, owing to the presence of chloride atoms in the crystal, the absolute configurations of C-2, C-17 and C-18 were assigned as 2*S*, 17*S* and 18*S* using the refinement of the Flack parameter [9]. On the basis of the above findings and the measurement of a positive optical rotation ${\left[\alpha \right]}_{D}^{25}$ = +12 (c 0.84, MeOH), the structure of **2a** was unambiguously elucidated and the trivial name (+)-decaryine A was proposed. (+)-Decaryine A is different from palustrine previously isolated from *Equisetum palustre* [10,11] with respect to the orientation of the spermidine moiety in the macrocyclic lactam.

**Table 2:** ^1^H and ^13^C-NMR data for compounds **2a** and **2b** in CDCl_3_.

	2a		2b	
	*δ*_H_ (*J* in Hz) ^a^	*δ*_C_	*δ*_H_	*δ*_C_^a^
2	3.56–3.62 m	56.5	3.71–3.73, m	57.05
3	2.22 dd (13.7, 2.3); 2.52–2.62 m	41.9	2.40 dd (14.4, 3.8); 2.56–2.61 m	40.4
4	-	173.2	-	173.9
5	7.45–7.53 m	-	8.10 br s	-
6	2.97–3.03 m; 4.12–4.21 m	37.4	3.05–3.11 m; 3.76–3.81 m	38.8
7	2.02–2.06 m; 2.42–2.52 m	26.9	1.98–2.03 m; 2.20–2.26 m	25.8
8	2.87–2.95 m; 3.27–3.36 m	46.3	3.05–3.11 m; 3.16–3.22 m	49.5
10	2.87–2.95 m; 3.21–3.27 m	45.8	2.88 ddd (13.2, 11.5, 2.4); 2.97–3.01 m	50.1
11	1.70–1.77 m; 1.94–2.02 m	22.5	1.67–1.77 m; 1.98–2.09 m	26.4
12	1.73–1.82 m	22.1	1.57–1.78 m	24.7
13	2.28–2.37 m; 2.87–2.93 m	54.4	2.46–2.53 m; 2.56–2.61 m	46.8
14	5.59 d (10.4)	126.6	5.55–5.58 m	126.95
15	5.79–5.84 m	123.7	5.83 ddd (10.0, 4.3, 3.7)	127.0
16	1.85–1.92 m; 2.28–2.37 m	20.5	1.83–1.85 m	22.2
17	2.73–2.79 m	63.5	3.16–3.22 m	57.0
18	3.53 dt (11.0, 1.8)	73.0	3.54, ddd (9.5, 6.4, 5.2)	72.2
19	1.61–1.77 m	28.9	1.50–1.56 m	26.7
20	1.13 t (7.4)	10.7	1.06 t (7.3)	10.4

^a^ Assignment of ^13^C-NMR data based on *J*-modulated, HSQC and HMBC spectra.

**Scheme 1:**
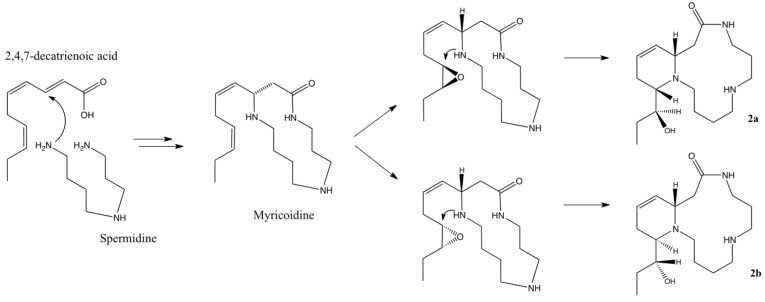
Potential biogenetic pathway for compounds **2a** and **2b**.

Compound **2b** (${\left[\alpha \right]}_{D}^{25}$ = −15 (c 0.13, MeOH)) was isolated as a yellow oil. The molecular formula was determined as C_17_H_31_N_3_O_2_ by HR-ESIMS (*m/z* 310.2497 [M+H]^+^). The ^13^C-NMR data (Table 2) in combination with analysis of HSQC and HMBC spectra revealed the presence of 17 carbons similar to those of compound **2**. These data along with ^1^H-NMR data (Table 2) and extensive analysis of COSY and HMBC correlations suggested that compound **2b** is a diastereoisomer of **2a**. The relative and absolute configurations of C-2, C-17 and C-18 could not have been assessed by spectroscopic analyses. Nevertheless, considering the fact that all spermidine alkaloids, including those described here, exhibit the same three-dimensional orientation at C-2 [7,12,13,14,15,16,17,18,19], we can speculate that the latter carbon is (*S*)-configurated. Schultz *et al*. [12] suggested that the first step of the biogenesis of spermidine alkaloids involves an enzyme-catalyzed Michael addition that would lead to the same stereochemistry at C-2 (Scheme 1). They also proposed a potential biogenetic pathway for palustrine [13] that can be adapted to the alkaloids of *A. decaryi* (Scheme 1). Indeed, a Michael addition of the primary amine of the butyl unit of spermidine to the α,β double bond of 2,4,7-decatrienoic acid, followed by amidification of the second primary amine may give a first intermediate, an alkaloid named myricoidine. Epoxidation of the double bond in the 17,18-position would then allow a nucleophilic attack by the nitrogen atom N-1 of the secondary amine at C-17 that induce the inversion of C-17 stereochemistry, with a concomitant 6-membered ring closure and hydroxyl group formation at C-18. In that case, we can postulate that epoxidation may occur on both faces of the double bond. Epoxide ring opening could then yield both (2*S*, 17*S*, 18*S*) and (2*S*, 17*R*, 18*R*) diastereoisomers as depicted in Scheme 1. Detection of dihydromyricoidine [19] in crude alkaloidal extract by MS analysis (see Supplementary Material) and notable difference in chemical shift of C-17 (*δ*_C_ 63.5 and *δ*_C_ 57.0 for compound **2a** and **2b**, respectively) are in agreement with this hypothesis. Therefore, the configuration of compound **2b**, named (–)-decaryine B, was proposed as 2*S*, 17*R*, 18*R*.

The known compounds lantibeside D (**3**) [20] and (*E*)-cinnamamide (**4**) [21] exhibited spectral data consistent with that reported. Finally, compounds **1**,**2a** and **3**,**4** were evaluated for their *in vitro* antiplamosdial activity. Compounds **1**, **2a** and **4** were not active (IC_50_ > 100 µM), whereas the lignan **3** exhibited a weak antiplasmodial activity (IC_50_ = 20.7 µM).

## 3. Experimental

### 3.1. General

IR spectra were taken on a Perkin Elmer FT-IR Paragon 1000. ^1^H and ^13^C-NMR spectra were recorded on a Brüker Avance 500 instrument in CDCl_3_ or MeOD. HR-ESIMS spectra were recorded on Waters GTC Premier and Waters LCT spectrometers. Single-crystal-X-ray diffraction analysis was performed on a Bruker AXS SMART APEX II diffractometer at low temperature (193(2)K) and using Mo K_α_ radiation (λ = 0.71073 Å). Optical rotations were measured with a Perkin Elmer 241 polarimeter. Column chromatographies were performed over Merck silica gel 60A (40–63 µm) and Sephadex^®^ LH-20. Analytical and semi-preparative TLC were performed on precoated Kieselgel 60 F_254_ plates (Merck, 0.5 mm and 20 × 20, 1 or 2 mm, respectively) using Dragendroff reagent for visualization. CCDC 923477 contains the supplementary crystallographic data for this paper. These data can be obtained free of charge via www.ccdc.cam.ac.uk/conts/retrieving.html (or from the CCDC, 12 Union Road, Cambridge CB2 1EZ, UK; fax: +44 1223 336033; e-mail: deposit@ccdc.cam.ac.uk).

### 3.2. Plant Material

*Androya decaryi* was collected twice by Frédéric Manjary (in 2008) and Claude Moulis (in 2009) in the Saint-Augustin region, in southwest Madagascar. Voucher specimens (ADFM2008 and ADCM2009) were deposited at the herbarium of the Institut Supérieur de Technologie of Toliara, Madagascar.

### 3.3. Extraction and Isolation

The dried and powdered leaves of a first sample of *A. decaryi* (108 g) were macerated with 100 mL of NH_4_OH (28% aqueous) and then successively percolated with dichloromethane (2 L) and MeOH (1 L). The CH_2_Cl_2_ extract was evaporated under reduced pressure at 40 °C to give 3.58 g of a crude extract which was dissolved in CH_2_Cl_2_ (200 mL), and extracted with HCl (0.5 M, 3 × 100 mL). The combined aqueous layers were then made alkaline by adding NaOH to pH 12 and extracted three times with CH_2_Cl_2_ (200 mL) to obtain the crude alkaloidal fraction (582 mg). This fraction was subjected to Si gel CC eluting with a CH_2_Cl_2_-MeOH gradient to yield seven fractions (F1–7). F7 (110 mg) was subjected to a second Si gel CC in the same conditions to afford Compound **1** (40 mg). F5 (150 mg) was rechromatographed over Si gel CC (CH_2_Cl_2_-MeOH gradient) and Sephadex LH 20 (CH_2_Cl_2_ then MeOH) to yield F521 (75 mg). Compound **2a** was repeatedly precipitated in MeOH/Et_2_O (1/1) from F_521_, and further crystallization of compound **2a** in CH_2_Cl_2_ afforded colorless crystal (34 mg).

The second sample of *A. decaryi* (1.5 kg) was extracted and partitioned according to the same procedure. A portion (2.8 g) of the crude alkaloidal fraction were further partitioned in a mixture of water (500 mL) and EtOAc (500 mL). The aqueous neutral layer was extracted thrice with EtOAc (500 mL) and the combined organic layers were concentrated under reduced pressure to yield AD2E1 (1 g). After alkalinization to pH 12 (28% NH_4_OH), the aqueous layer was extracted with EtOAc (3 × 500 mL) to give after concentration 1.5 g of AD2E2. A white solid precipitated in CH_2_Cl_2_ from AD2E1 to afford after filtering (*E*)-cinnamamide **4** (125 mg). The resulting filtrate was further concentrated (880 mg) and fractionated on Si gel CC (CH_2_Cl_2_-MeOH gradient) to yield compound **3** (30 mg). AD2E2 was subjected to combinations of Sephadex LH 20 (CH_2_Cl_2_), Si gel (CH2Cl2-MeOH gradient) chromatography and final semi-preparative TLC (CH_2_Cl_2_/MeOH/NHEt_2_ 9/0.5/0.5) yielding compound **2b** (1.3 mg).

*(–)-(2S)-2-Phenyl-1,5,9-triazacyclotridecan-4-one* (**1**). Yellow oil; ${\left[\alpha \right]}_{D}^{25}$ = −13 (c 3.0, MeOH); IR (film) *ν*_max_ 3420, 3250, 3075, 2920, 1639; ^1^H and ^13^C-NMR see Table 1; ESI-MS positive *m/z* [M+H]^+^ 276 (100); HR-ESIMS *m/z* [M+H]^+^ 276.2078 (calc for C_16_H_26_N_3_O, 276.2076).

*Decaryine A* (**2a**). Colorless crystals; ${\left[\alpha \right]}_{D}^{25}$ = +12 (c 0.84, MeOH); IR (film) *n*_max_ 3383, 3060, 2926, 1648, 1093; ^1^H and ^13^C-NMR see Table 2; ESI-MS positive *m/z* [M+H]^+^ 310 (100); HR-ESIMS *m/z* [M+H]^+^ 310.2501 (calc for C_17_H_32_N_3_O_2_, 310.2495).

*Decaryine B* (**2b**). Yellow oil; ${\left[\alpha \right]}_{D}^{25}$ = −15 (c 0.13, MeOH); ^1^H and ^13^C-NMR see Table 2; ESI-MS positive *m/z* [M+H]^+^ 310 (100); HR-ESIMS *m/z* [M+H]^+^ 310.2497 (calc for C_17_H_32_N_3_O_2_, 310.2495).

*Lantibeside D* (**3**). White amorphous powder; ${\left[\alpha \right]}_{D}^{25}$ = +9 (c 2.0, MeOH); ^1^H and ^13^C-NMR see [20]; ESI-MS positive *m/z* [M+Na]^+^ 541 (100), [2M+Na]^+^ 1059 (63).

### 3.4. Antiplasmodial Bioassay

Parasites (FCM-29 strain) were cultured according to the method described by Trager and Jensen [22] with modifications described by Benoit *et al.* [23]. The cultures were synchronized every 48 h by 5% D-sorbitol lysis [24] (Merck, Darmstadt, Germany). The FCM-29 strain was considered as a chloroquine-resistant strain (chloroquine IC_50_: 145 nM). *In vitro* antimalarial activity testing was performed by [3H]-hypoxanthine (Amersham, Orsay, France) incorporation as described by Desjardins *et al*. [25] with modifications [26].

## 4. Conclusions

The phytochemical investigation of *Androya decaryi* led to the isolation of three macrocyclic spermidine alkaloids named (–)-(2*S*)-2-phenyl-1,5,9-triazacyclotridecan-4-one (**1**), (+)-decaryine A (**2a**) and (–)-decaryine B (**2b**). To the best of our knowledge, Compound **1** was previously synthesized, but it is reported here for the first time as a natural product. This is also the first report of spermidine alkaloids in Scrophulariaceae. The absolute configurations of alkaloids **1** and **2a** were unambiguously elucidated, supporting the hypothesis described by Hesse and co-workers of a common initial biogenetic step. A similar biogenetic pathway was then postulated for compound **2a** and **2b**, allowing us to propose an absolute configuration for (–)-decaryine B (**2b**). The structural complexity of macrocyclic spermidine alkaloids has stimulated numerous syntheses, especially to unambiguously determine their absolute configuration [12,13,18,19,27,28]. Thus, enantioselective synthesis of (–)-decaryine B would confirm these hypotheses. Evaluation of their antiplasmodial properties showed that compounds **1**, **2a**, **3** and **4** exhibited no significant activity against *Plasmodium falciparum*.

## Supplementary Materials

Supplementary materials can be accessed at: http://www.mdpi.com/1420-3049/18/4/3962/s1.
