# Supplementary material for: Macrocyclic Spermidine Alkaloids from Androya decaryi L. Perrier
Source: Molecules. 2013 Apr 4;18(4):3962–71. doi: 10.3390/molecules18043962 (PMC6269681; doi:10.3390/molecules18043962)

# Supplementary Materials

**Figure S1.** $^{1}$H-NMR spectrum of decaryine **2a** ($CDCl_3$, 500 MHz).

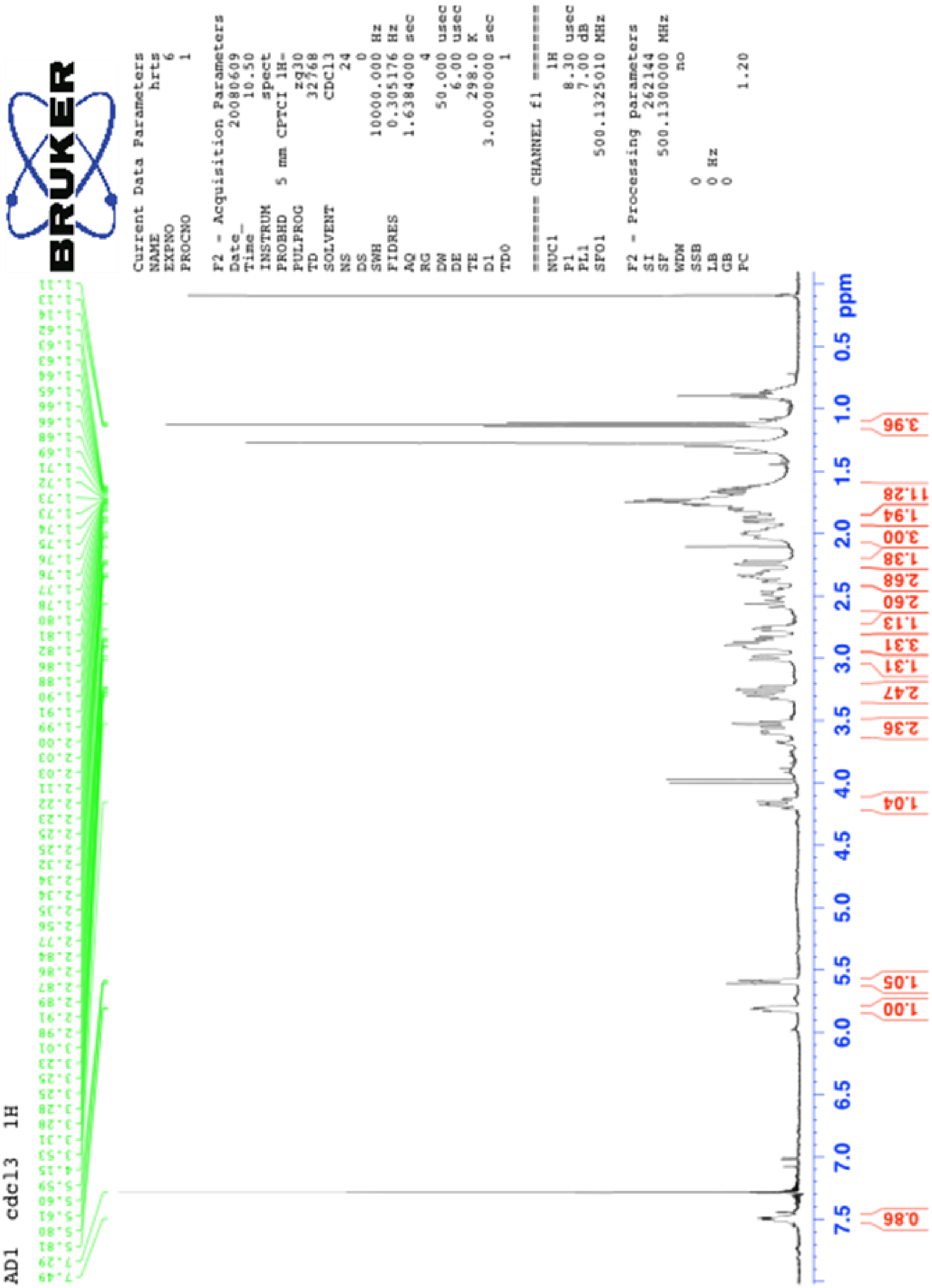

**Figure S2.** *J*-modulated $^{13}C$-NMR spectrum of decaryine **2a** ($CDCl_3$, 125 MHz).

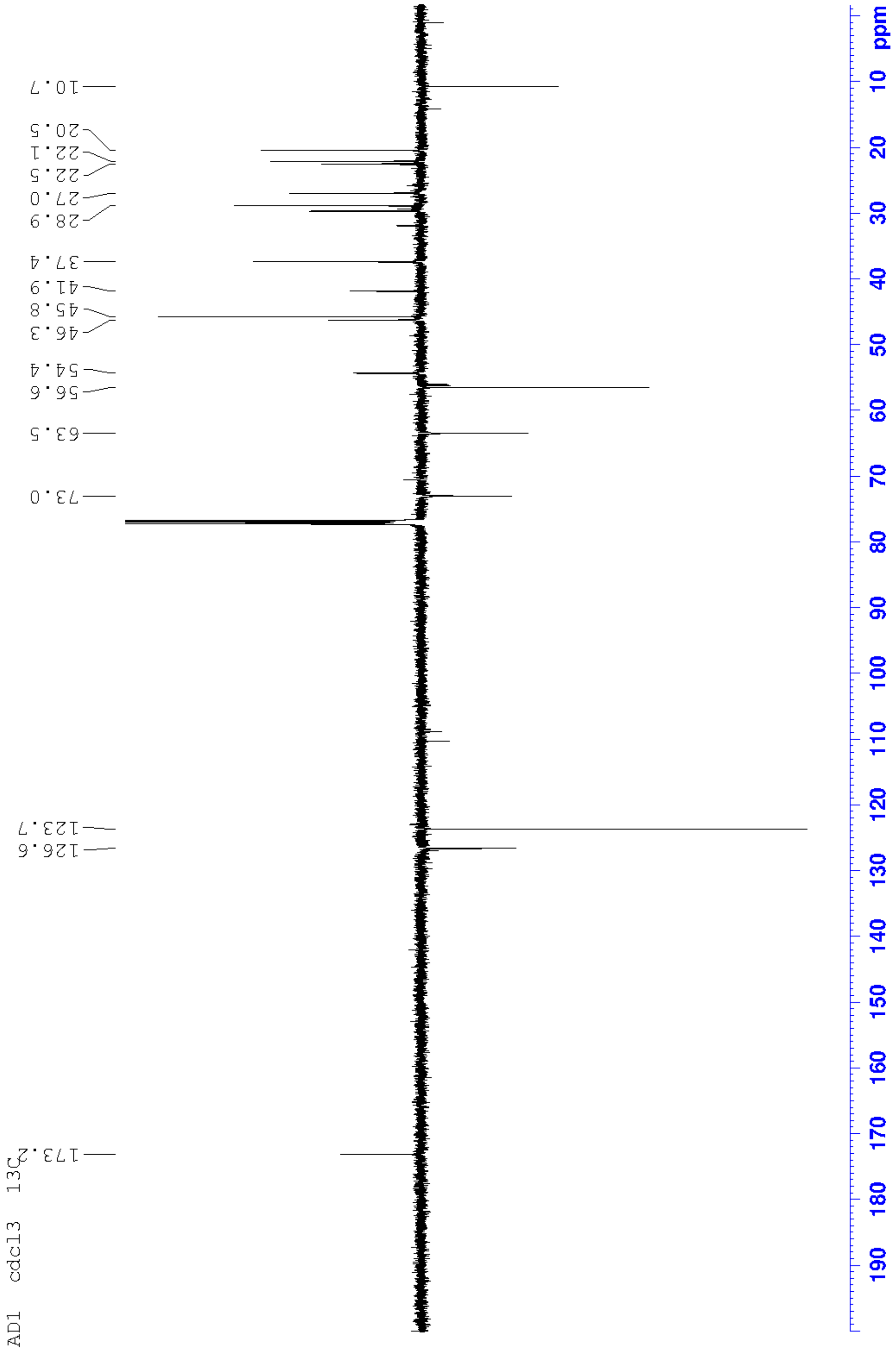

**Figure S3.** $^{1}$H-NMR spectrum of decaryine **2b** ($CDCl_3$, 500 MHz).

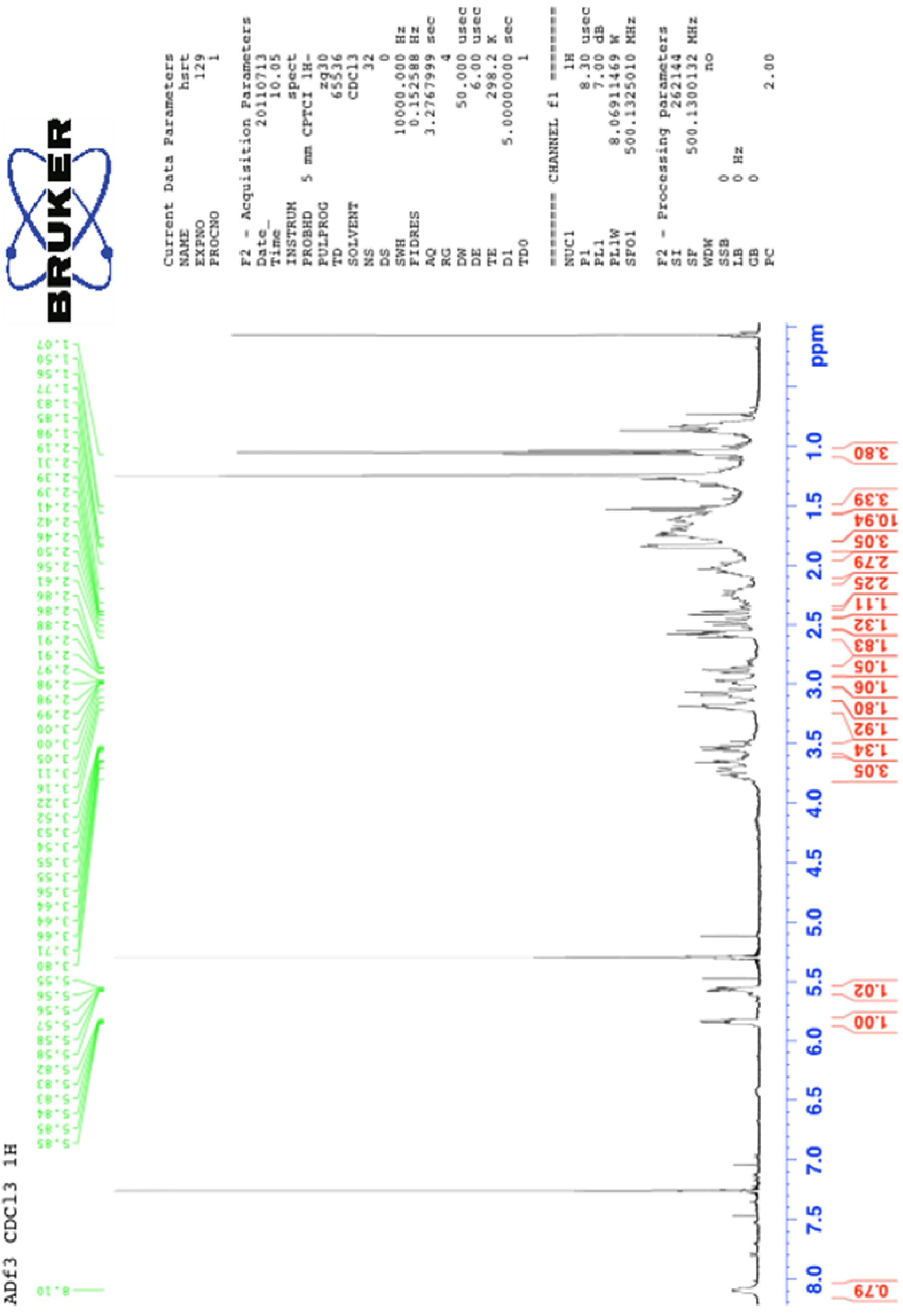

**Figure S4.** *J*-modulated $^{13}$C-NMR spectrum of decaryine **2b** ($CDCl_3$, 125 MHz).

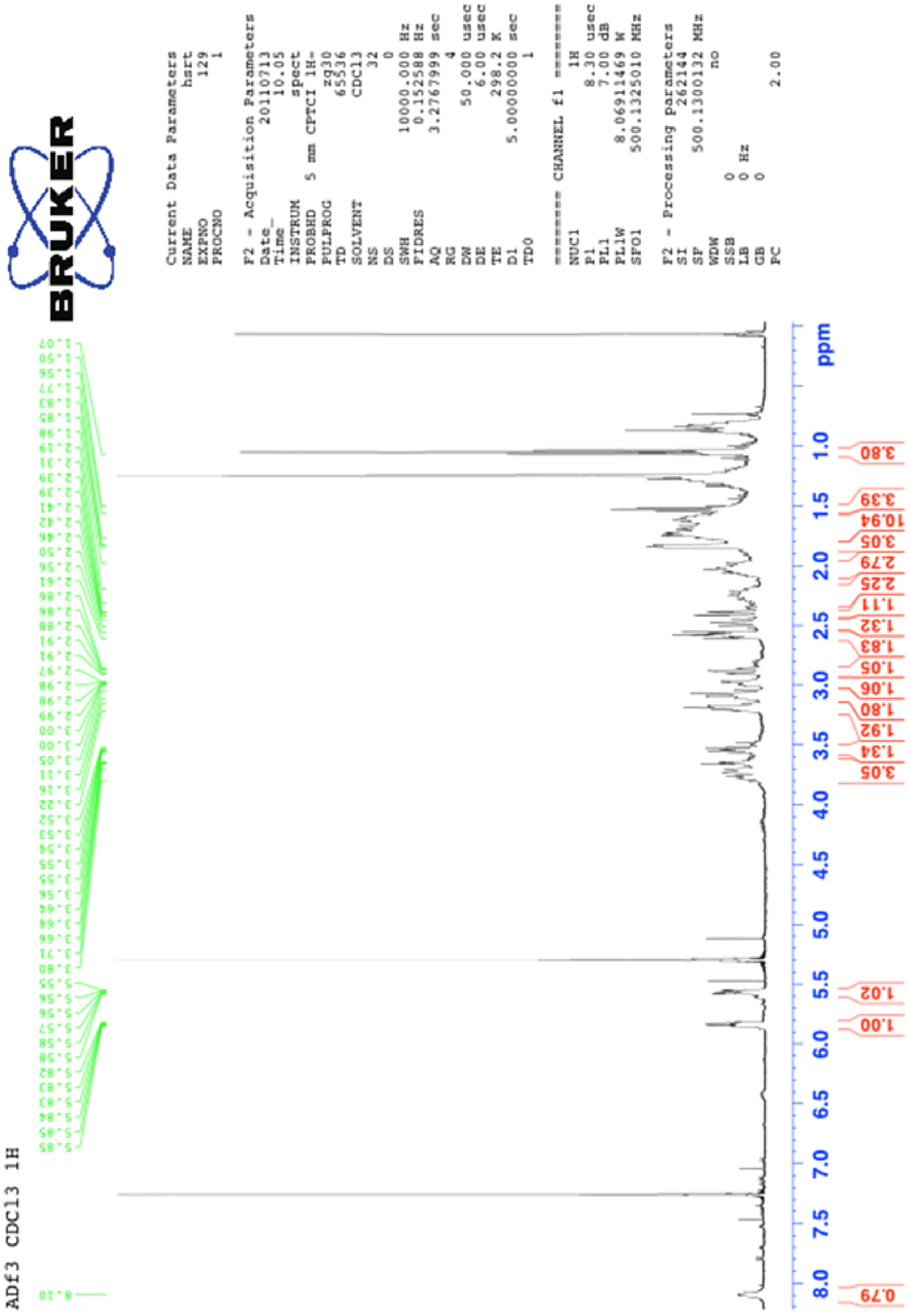

**Figure S5.** Identification of dihydromyricoidine by MS fragmentations analysis.

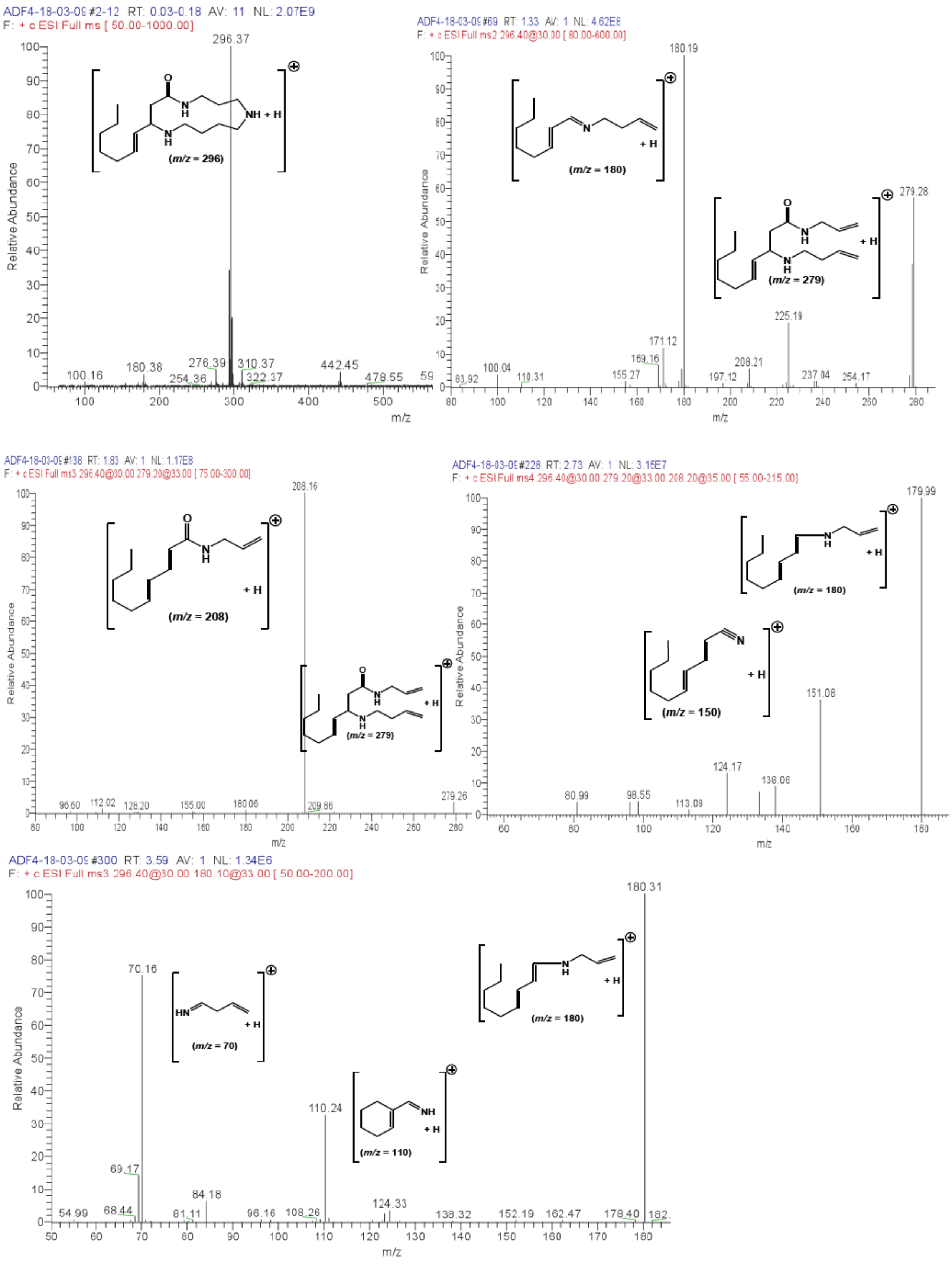

Supplement: Supplementary file 1 [file molecules-18-03962-s001.pdf]
